# Population Pharmacokinetics and Dosage Optimization of Teicoplanin in Children With Different Renal Functions

**DOI:** 10.3389/fphar.2020.00552

**Published:** 2020-05-05

**Authors:** Liuliu Gao, Hua Xu, Qi Ye, Sichan Li, Jun Wang, Yan Mei, Changhe Niu, Ting Kang, Chen Chen, Yang Wang

**Affiliations:** ^1^Department of Clinical Pharmacy, Wuhan Children’s Hospital (Wuhan Maternal and Child Healthcare Hospital), Tongji Medical College, Huazhong University of Science and Technology, Wuhan, China; ^2^Department of Neonatology, Wuhan Children’s Hospital (Wuhan Maternal and Child Healthcare Hospital), Tongji Medical College, Huazhong University of Science and Technology, Wuhan, China; ^3^Department of Pharmacy, Union Hospital, Tongji Medical College, Huazhong University of Science and Technology, Wuhan, China

**Keywords:** teicoplanin, population pharmacokinetics, Chinese children, children with different renal functions, dosing optimization

## Abstract

**Objective:**

The purposes of our study were to investigate the population pharmacokinetics of teicoplanin in Chinese children with different renal functions and to propose the appropriate dosing regimen for these pediatric patients.

**Methods:**

We performed a prospective pharmacokinetic research on children aged 0–10 years, with different renal functions. The population pharmacokinetics model of teicoplanin was developed using NLME program. The individualized optimal dosage regimen was proposed on the basis of the obtained population pharmacokinetics parameters.

**Results:**

To achieve the target trough level of 10–30 mg/L, optimal dosing regimen for children with different renal functions are predicted as follows based on the population PK simulations: children with moderate renal insufficiency need three loading doses of 6 mg/kg q12h followed by a maintenance dose of 5 mg/kg qd; children with mild renal insufficiency require three loading doses of 12 mg/kg q12h followed by a maintenance dose of 8 mg/kg qd; children with normal or augmented renal function should be given three loading doses of 12 mg/kg q12h followed by a maintenance doses of 10 mg/kg qd.

**Conclusion:**

The first study on the population pharmacokinetics of teicoplanin in Chinese children with different renal functions was performed. Individualized dosing regimen was recommended for different renal function groups based on population PK model prediction.

## Introduction

Teicoplanin is a glycopeptide antibacterial which was approved in Italy and France since 1980s. Now, it is utilized in at least 60 countries to treat diseases caused by Gram-positive infections (Wilson et al., 1994; Ogami et al., 2019) through inhibiting the synthesis of cell-wall peptidoglycans of the bacterial (Chinese Society of Hematology et al., 2016). Comparing with vancomycin, the structure of teicoplanin is different through adding a fatty acid side chain on the peptide skeleton, improving its lipophilicity and making it easier to penetrate the organization (Wang et al., 2018).

Teicoplanin PK is time-dependent and has a long half-life of elimination (30–180 h) (Harding and Sorgel, 2000; Hagihara et al., 2012; Boztug et al., 2017; Zhou et al., 2018), which results in a great individual variability, particularly in children (Yamada et al., 2012). The serum trough concentration of teicoplanin is closely related to its therapeutic efficacy (Niwa et al., 2010; Ahn et al., 2011; Seki et al., 2012). Therefore, the dosing regimen should be adjusted based on the trough level of teicoplanin (Hagihara et al., 2012; Hu et al., 2018). According to previous researches, 10–30 mg/L was regarded as the target teicoplanin trough level for successful treatment (Hiraki et al., 2015; Nakamura et al., 2015; Ueda et al., 2016; Zhou et al., 2018) and therapeutic drug monitoring (TDM) is an effective method to assure the adequate trough concentration for therapy (McKenzie, 2011; Strenger et al., 2013; Yamada et al., 2017). Teicoplanin is primarily excreted by kidney, and renal function is more likely to affect its pharmacokinetic (Wilson, 2000). A few researches suggested that, in order to reach the target level, dose adjustment of teicoplanin on the basis of population pharmacokinetics parameters and software supporting TDM is required (Niwa et al., 2010; Ogami et al., 2019).

To ensure the safety and efficacy of teicoplanin in children, dosage regimen design based on its pharmacokinetic/pharmacodynamic (PK/PD) characteristics is required (Allegaert et al., 2013; Goulenok and Fantin, 2013). Although several studies have assessed the pharmacokinetic of teicoplanin (Ogawa et al., 2013; Cazaubon et al., 2017; Wang et al., 2018), there are no researches performed in Chinese children with different renal functions. Meanwhile, both the pharmacokinetic profile of teicoplanin and the dosing regimen achieving a PK/PD target in Chinese children have not been completely characterized. Inappropriate dosage for children will result in an ineffective treatment or antibiotic resistance with this drug (Byrne et al., 2017). Therefore, the aims of our work were to investigate the population pharmacokinetics of teicoplanin in Chinese children with different renal functions and to propose optimal dosage regimens for them.

## Materials and Methods

### Study Population

The population pharmacokinetics study of teicoplanin was a prospective research, which was performed at Wuhan Children’s hospital from February 2016 to January 2019. Children aged 0–10 years infected by Gram-positive bacterial and received teicoplanin treatment were included. Children were excluded if they were registered in other trials, without complete dosing information, or intolerant to teicoplanin treatment.

The studies involving human participants were reviewed and approved by the Ethics Committee of Wuhan Children’s hospital. The guardians provided written informed consent for their children to participate in this study.

### Dosage Regimen and Pharmacokinetic Sampling

Teicoplanin produced by Sanofi-Aventis was given through intravenous infusion. The dosage regimen was carried out as three loading doses of 10 mg/kg q12h followed by a maintenance dose of 10 mg/kg qd for children with different renal functions. The dosage could be adjusted according to the clinical condition of patients. The number of samples collected from per patient was 1–3. The dosing, infusion, and sampling time were accurately recorded respectively. During the whole teicoplanin treatment period, serum samples were collected and centrifuged for 10 minutes, and teicoplanin concentrations were determined by employing the high-performance liquid chromatography (HPLC) method. The following individual laboratory and demographic parameters were collected respectively, including age, gender, height, weight (WT), serum creatinine concentration (SCR), blood urea nitrogen (BUN), serum cystatin C (Cys-C), uric acid (UA), alanine aminotransferase (ALT), aspartate aminotransferase (AST), total bilirubin (TBIL), and γ-glutamyltranspeptidase (γ-GT). Serum creatinine assay applied the enzymatic method as reported in previous studies using Roche cobas 8000 c702. Estimated glomerular filtration rate (eGFR) was obtained by employing the modified Schwartz formula [eGFR(ml/min·1.73m^2^) = 0.413*(Height/Serum creatinine)] (Schwartz et al., 2009).

### Analytical Method of Teicoplanin

The concentration of teicoplanin was determined by employing the HPLC method (Agilent Technologies Inc., 1260 infinity). The steps of serum sample preparation were as follows: 0.5-ml serum sample was added into the solid-phase extraction column (Agela Technologies, Cleanert ODS C18), and then eluted using 50% acetonitrile. The Innoval C18 column (Agela Technologies, 10 μm, 100 Å, 4.6 × 250 mm) was used to achieve the separation. Sodium dihydrogen phosphate (0.01 mmol/L): acetonitrile = 75:25 (PH = 3.3) was used as the mobile phase. The wavelength of ultraviolet (UV) detection was set as 215 nm. The linear range of teicoplanin detection was 2.0–180 mg/L, with limits of detection of 2.0 mg/L. Both the intra- and inter-day precisions were within 10%.

### Population Pharmacokinetics Modeling

The study of population pharmacokinetics was performed by applying the modeling program Phoenix^®^ NLME (Version 8.1, Pharsight Corporation, USA) and R program (Version 3.5.1). The pharmacokinetic parameters and their variability were estimated by first order conditional estimation-extended least squares method.

The population pharmacokinetics model was composed of a structural model and several random effect models. The structural model was used to illustrate the relationship between concentration and time, the random effect models was applied to evaluate the inter- and intra-individual variability of population pharmacokinetics. Both one- and two-compartment structural models with first-order elimination were evaluated. The residual-variability model was chosen based on changes of the objective function value (OFV, −2 * log-likelihood) and visual diagnostic plots.

The exponential model was applied to describe inter-individual variability, which was shown as Eq. 1:

Eq. 1 $$P_{i}=\theta *\exp(\eta_{i})$$

in which *P_i_* is the estimated parameter value of the individual *i*, *θ* represents the typical population parameter, and *η_i_* is assumed to be normally distributed with mean 0 and variance ω^2^ as diagonal matrixes.

The intra-individual variability of the pharmacokinetic parameters was evaluated by employing the additive, proportional, combined additive or power model, respectively, which were usually assessed for residual unexplained variability (RUV) model and were shown as follows:

Eq. 2 Y=IPRED+ϵ

Eq. 3 Y=IPRED×exp(1+ϵ)

Eq. 4 $$Y=\mathrm{IPRED}\times \exp(1+\epsilon_{1})+\epsilon_{2}$$

Eq. 5 Y=IPRED+IPRED^power×ϵ

where *Y* represents the observed serum teicoplanin concentration, *IPRED* is the individual prediction, *ϵ_n_* is regarded as following a Gaussian distribution with mean 0 and variance σ^2^ as diagonal matrixes.

### Covariate Analysis

Before covariate analysis, we analyzed the correlation between the covariates to avoid including co-linear variables in the model. In order to evaluate the effect of each variable on population pharmacokinetic parameters, the likelihood ratio test was employed, in which demographic characteristics (including age, weight, height, and BSA), hepatic functions (TBIL, AST, and ALT) and renal functions (BUN, UA, SCR, and eGFR) were all included.

Because the maturation development of children has a great impact on clearance (CL), four different models based on allometric scaling were tested using Eqs. 6–10:

Eq. 6 $$\mathrm{CL}=\mathrm{TV}(\mathrm{CL})\times {\left(\frac{\mathrm{WT}}{WT_{\mathrm{median}}}\right)}^{k_{1}}\times \mathrm{MF}$$

In which *WT_median_* represents the median of weight. *MF* is the fraction of the population median value of CL. *k_1_* represents the exponent co-efﬁcient of WT;

Model I: The simplest exponent model, *MF* was fixed to 1 and the exponent k_1_ was estimated.

Eq. 7 $$\mathrm{CL}=\mathrm{TV}(\mathrm{CL})\times {\left(\frac{\mathrm{WT}}{WT_{\mathrm{median}}}\right)}^{k_{1}}$$

Model II: The maturation model, the exponent *k_1_* was fixed to 0.75. *MF* was calculated as follows:

Eq. 8 $$\mathrm{MF}=\frac{1}{1+{\left(\frac{\mathrm{Age}}{TM_{50}}\right)}^{-\gamma }}$$

where *TM_50_* represents the age at which maturation achieves 50% of the population median CL. γ represents the Hill coefficient that was utilized to define the steepness of the sigmoid decrease.

Model III: The WT-dependent exponent model:

Eq. 9 $$k_{1}=k_{0}-\frac{k_{\max}\times WT^{\gamma }}{k_{50}^{\gamma }+WT^{\gamma }}$$

Model IV: The age-dependent exponent model:

Eq. 10 $$k_{1}=k_{0}-\frac{k_{\max}\times {\mathrm{Age}}^{\gamma }}{k_{50}^{\gamma }+{\mathrm{Age}}^{\gamma }}$$

In which *k_0_* is the exponent value when the theoretical WT is 0 kg (Eq. 9) or the theoretical age is 0 years (Eq. 10), *k*_max_ represents the maximum decrease value of the exponent, *k*_50_ represents the WT (Eq. 9) or age (Eq. 10) at which a 50% drop in the maximum decrease is achieved.

The variables inclusion forms of the previous study (Huang et al., 2019) were presented as follows, including continuous variables (Eqs. 11–13) and categorical variables (Eq. 14):

Eq. 11 $$P_{i}=\mathrm{TV}(P)+\theta \times \frac{\mathrm{COV}}{{\mathrm{COV}}_{\mathrm{median}}}$$

Eq. 12 $$P_{i}=\mathrm{TV}(P)+\theta \times (\mathrm{COV}-{\mathrm{COV}}_{\mathrm{median}})$$

Eq. 13 $$P_{i}=\mathrm{TV}(P)\times {\left(\frac{\mathrm{COV}}{{\mathrm{COV}}_{\mathrm{median}}}\right)}^{\theta }$$

Eq. 14 $$P_{i}=\mathrm{TV}(P)\times \exp(\theta )$$

where *θ* represents the influence degree of covariate on the parameters, *COV* is the individual covariate value, *COV_median_* represents the median value of the covariate.

The OFV, Akaike information criteria (AIC), and Bayesian information criteria (BIC) were employed in the selection of the competing non-nested aforementioned models, and models with the lowest values of OFV, AIC, and BIC were regarded as superior.

During the process of population pharmacokinetic modeling, both forward and backward selections were utilized and the covariates selected or excluded depended on the value changes of OFV as previously reported (Zhao et al., 2015). In the forward selection, when the decrease in OFV was more than 3.84 points (P < 0.05, df = 1), the covariate would be added into the basic model to build a integral model. Then, the backward selection was applied to reassess the importance of the covariates. The covariates should be removed if the increase of OFV was less than 6.64 (P < 0.01, df = 1). Finally, the final population pharmacokinetics model was constructed.

### Validation of Final Population Pharmacokinetics Model

To evaluate the final population pharmacokinetics model and the parameters, goodness-of-fit plots, normalized prediction distribution errors (NPDE), nonparametric bootstrap, and visual predictive check (VPC) were employed. Goodness-of-fit plots were initially applied to evaluate the accuracy of model prediction, employing plots of observed concentrations against individual or population predictions and conditional weighted residuals (CWRESs) against time or population predictions, respectively. Nonparametric bootstrap was employed to estimate the performance and stability of the final model. One thousand replicated datasets generated from random sampling with replacement were evaluated. The 95% confidence interval (95% CI) and the median of the final parameters were calculated and compared with the final parameters estimated by NLME program.

NPDE was simulated for 1,000 times and the results were generalized graphically by default as obtained from the R package, including Quantile-quantile plot and the NPDE histogram. NPDE is expected to follow normal distribution. VPCs of children with different renal functions were carried out and the obtained datasets were simulated for 1,000 times.

### Simulation and Dosing Optimization

To investigate the optimal dosage regimen, the obtained population pharmacokinetics parameters were employed to perform the Monte Carlo simulation, which was applied to simulate concentration-time curves after multiple teicoplanin doses in children with different renal functions: I. moderate renal insufficiency (eGFR: 30–60 ml/min·1.73m^2^); II. mild renal insufficiency (eGFR: 60-90 ml/min·1.73m^2^); III. normal renal function (eGFR: 90–130 ml/min·1.73m^2^); IV. augmented renal function (eGFR: larger than 130 ml/min·1.73m^2^). The therapeutic results were closely associated with teicoplanin trough concentration (Seki et al., 2012) and the trough concentration of 10–30 mg/L was applied as an effective therapeutic target. Concentration-time curves of different dosage regimens in children with different renal functions were simulated based on the final population pharmacokinetics parameters. The optimal dosing regimens were finally selected according to the status of trough levels distributed within the target concentration range after administration.

## Results

### Study Population

A total of 136 patients were recruited for population pharmacokinetics study and all of them completed the teicoplanin treatments. The patients participated in the study were Chinese children aged 0.09 to 9.42 years and consisted of 79 males and 57 females. The numbers of children with augmented renal function, normal renal function, mild renal insufficiency, and moderate renal insufficiency were 42, 63, 23, and 8, respectively. The clinical characteristics of patients were shown in **Table 1**. In our study, there was no neonate and the youngest patient is 2 months. There are 48 cases of infants under 1 year old with eGFR of 98.08 ± 27.28 ml/min·1.73m^2^ and 42 cases of 1–2 years old infants with eGFR of 120.70 ± 33.65 ml/min·1.73m^2^.

**Table 1:** Baseline characteristics of children for population pharmacokinetics modeling (n = 136, Mean ± SD).

	Number	Mean ± SD	Median (Range)
Patients	136		
Gender(M:F)	79:57		
Age (years)		2.19 ± 2.25	1.25 (0.17–9.42)
WT (kg)		12.12 ± 6.34	10 (3.5–38)
Height (cm)		80.90 ± 20.79	80 (52–145)
BSA (m^2^)		0.52 ± 0.21	0.4 (0.22–1.43)
Laboratory parameter			
BUN (mmol/L)		3.51 ± 1.75	3.26 (0.8–10.1)
SCR (μmol/L)		30.05 ± 20.42	25.9 (13.2–172.1)
UA (μmol/L)		227.51 ± 99.94	208.85 (53.2–588)
eGFR (ml/min·1.73m^2^)		116.92 ± 38.45	118.99 (30.09–280)
TBIL (μmol/L)		10.82 ± 19.06	6.55 (2–150.6)
ALT (U/L)		56.33 ± 145.34	23 (6–1,000)
AST(U/L)		93.57 ± 130.32	50.5 (14–750)

eGFR was calculated with modified Schwartz formula. BSA were calculated using the Mosteller formula: BSA (m^2^) = {(height [cm] * weight [kg])/3,600}^1/2^.

### Population Pharmacokinetics Modeling

**Figure 1:**
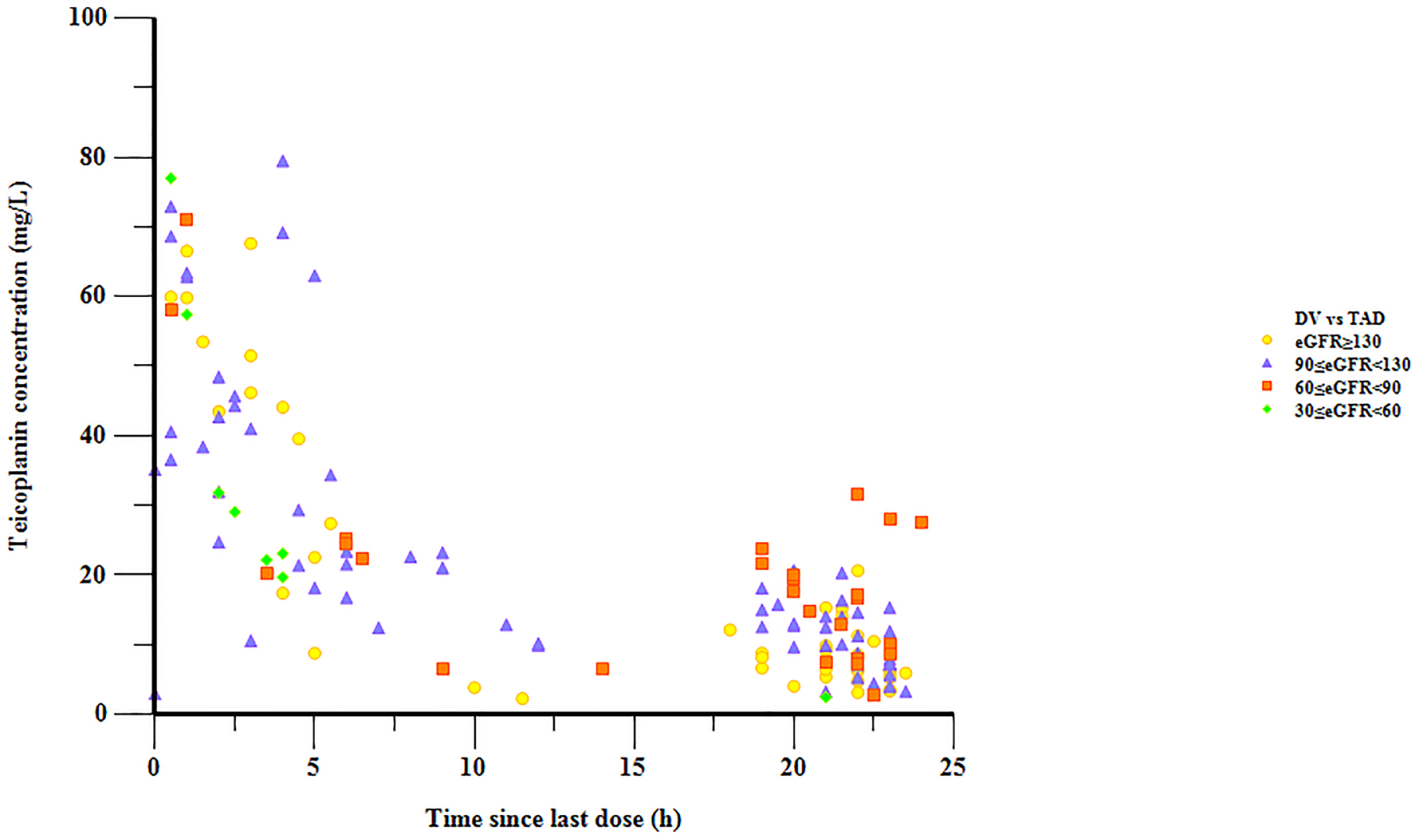
Teicoplanin concentrations versus time since the last dose.

A total of 155 teicoplanin concentrations in the range of 2.22 to 79.49 mg/L were obtained for population pharmacokinetics modeling. The number of samples collected from per patient was 1–3. Finally, of the 155 serum concentrations detected, 150 were the steady-state concentrations (96.77%), 23 were the peak concentrations (14.84%), and 52 were trough concentrations (33.55%). The numbers of samples collected from patients of the four different groups including the augmented renal function group, normal renal function group, mild renal insufficiency group, and moderate renal insufficiency group were 49, 70, 28, and 8, respectively. The concentrations collected from each renal function subgroup were plotted in different colors in **Figure 1**.

**Figure 2:**
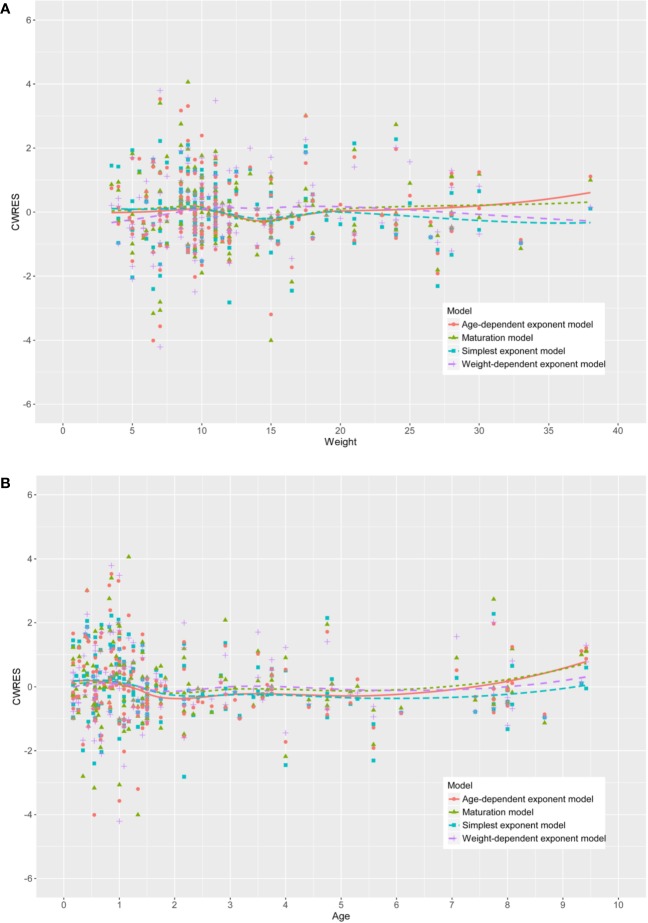
The conditional weighted residuals (CWRESs) with **(A)** weight or **(B)** age of the four development models.

The population pharmacokinetics characteristics of teicoplanin could be best illustrated using a two-compartment model with first-order elimination, which was parameterized as central volume of distribution (V_1_), peripheral volume of distribution (V_2_), inter-compartment clearance (Q), and clearance (CL). The result of the selection of RUV model suggested that the power model (Eq. 5) was the best fit with the power value of 0.5. As can be seen in the figure in **Supplementary Material**, the correlation coefficient between covariates higher than 0.5 were considered as a significant correlation and were not included in covariates selection. As shown in **Table 2**, to account for WT and age, four physical maturation models for CL were evaluated. In the forward inclusion step, by exploring the relationship of apparent total CL with WT and age, the simplest allometric model (**Model I**) had the lowest OFV, AIC, and BIC values and showed the best fit. In addition, as shown in **Figure 2**, the CWRESs with WT or age of the four physical maturation models have no significant change trend. The loess curves obtained from locally weighted regression were approximately parallel to the horizontal line. **Table 3** presented a summary of the pharmacokinetic model development process according to decreasing order of OFV. The decreases in OFV of WT and eGFR on CL were 53.30 points and 33.79 points, respectively, suggesting that both weight and eGFR exhibited significant impacts on teicoplanin CL. In the final model, the equations to derive the population values for V_1_, V_2_, Q, and CL are as follows:

**Table 2:** Parameter estimates of the four physical maturation clearance candidate models.

Parameters	Model I: the simplest exponent model	Model II: the maturation model	Model III: the WT-dependent exponent model	Model IV: the age-dependent exponent model
OFV	1,044.19	1,059.15	1,062.80	1,060.77
AIC	1,070.22	1,091.15	1,102.80	1,100.77
BIC	1,109.78	1,139.85	1,163.67	1,161.64
MF = 1/[1 + (Age/TM_50_)^–γ^]				
TM_50_ (SE%)	–	1.25 (20.80)	–	–
γ (SE%)	–	0.33 (26.21)	–	–
k_1_ = k_0_ – k_max_ × WT^γ^/(k_50_^γ^ + WT^γ^) or k_1_ = k_0_–k_max_ × Age^γ^/(k_50_^γ^ + Age^γ^)				
k_0_ (SE%)	–	–	0.35 (12.58)	0.60 (15.67)
k_max_ (SE%)	–	–	1.12 (12.50)	1.05 (13.15)
k_50_ (SE%)	–	–	4.12 (12.27)	0.73 (21.18)
γ (SE%)	–	–	−0.89 (12.42)	−0.23 (26.61)

MF, the fraction of the population median value of clearance; TM_50_, the age at which maturation achieves 50% of the population median clearance; γ, Hill coefﬁcient determining the steepness of the sigmoidal decline; k_1_, exponent coefﬁcient of WT; k_0_, the exponent value when theoretical WT is 0 kg or age is 0 year; k_50_, the WT or age at which there is a 50% decrease in the maximum decrease; k_max_, maximum decrease of the exponent; SE(%), percent standard error.

**Table 3:** Final model development process and statistical analysis.

step	Covariates screening	OFV	ΔOFV	*P* value	Comments
1	none	1,096.49			Base model
	forward inclusion				
2	CL-WT	1,044.19	−52.30	<0.01	
3	CL-WT-eGFR	1,010.40	−33.79	<0.01	
4	CL-WT-eGFR/V_2_-WT	997.52	−12.88	<0.01	
5	CL-WT-eGFR/V_2_-WT/V_1_-lnWT	987.87	−9.65	<0.01	
6	CL-WT-eGFR/V_2_-WT/V_1_-lnWT/Q-WT	982.58	−5.29	<0.05	Full model
	backward elimination				
7	CL-WT-eGFR/V_2_-WT/V_1_-lnWT	987.87	5.29	>0.01	Final model

ΔOFV, the change of OFV.

Eq. 15 $$V_{1}(L)=\theta_{V_{1}}\times {\left(\frac{\ln WT}{2.3}\right)}^{\theta_{1}}$$

Eq. 16 $$V_{2}(L)=\theta_{v_{2}}\times {\left(\frac{\mathrm{WT}}{10}\right)}^{\theta_{2}}$$

Eq. 17 $$\mathrm{CL}(L/h)=\theta_{\mathrm{CL}}\times {\left(\frac{\mathrm{WT}}{10}\right)}^{\theta_{3}}\times {\left(\frac{\mathrm{eGFR}}{118.99}\right)}^{\theta_{4}}$$

Eq. 18 $$Q(L/h)=\theta_{Q}$$

in which WT is given in kilogram.

**Table 4** showed the parameters and bootstrap confidence intervals for the final model. The typical values of the population pharmacokinetic parameters obtained from the final model were as follows: V_1_ = 2.31 L, V_2_ = 16.19 L, CL = 0.13 L/h, Q = 0.23 L/h, which were normalized by the median WT and median eGFR. The bootstrap analysis suggested that it was very similar between the estimated parameters and the median of the bootstrap replicates (relative error < 10%) and the former laid in 95% CI of the latter.

**Table 4:** Parameter estimates and bootstrap results of the final model.

Parameter	Final model		Bootstrap analysis			Bias (%)
	Estimate	SE(%)	2.5th percentile	Median Estimate	97.5th percentile	
θ_V1_(L)	2.31	13.31	1.51	2.16	2.74	−6.49
θ_V2_(L)	16.19	3.10	12.34	15.25	18.22	−5.81
θ_CL_(L/h)	0.13	21.51	0.04	0.13	0.23	0
θ_Q_(L/h)	0.23	13.13	0.10	0.24	0.37	4.35
θ_1_	0.14	30.69	0.02	0.15	0.29	7.14
θ_2_	0.19	30.68	0.02	0.20	0.41	5.26
θ_3_	0.74	29.67	0.51	0.81	1.85	1.46
θ_4_	0.60	30.49	0.12	0.58	1.01	−3.33
Inter-individual						
ω_V1_(%)	105.43	5.95	88.84	104.52	120.20	0.86
ω_V2_(%)	19.58	6.23	17.46	20.40	23.34	4.19
ω_CL_(%)	44.67	5.88	38.44	44.32	50.21	−0.78
ω_Q_(%)	42.86	5.95	38.81	46.65	54.49	8.84
η_V1_-shrinkage(%)	29.81					
η_V2_-shrinkage(%)	67.67					
η_CL_-shrinkage(%)	29.23	–	–	–	–	–
η_Q_-shrinkage(%)	47.96					
Residual variability						
σ	0.46	30.20	0.19	0.48	0.77	4.35
ϵ-shrinkage(%)	19.71	–	–	–	–	–

θ_V1_, typical value of central volume of distribution; θ_V2_, typical value of peripheral volume of distribution; θ_CL_, typical value of apparent clearance; θ_Q_, typical value of inter-compartment clearance; θ_1_, exponent for lnWT as covariate for V_1_; θ_2_, exponent for WT as covariate for V_2_; θ_3_, exponent for WT as covariate for CL; θ_4_, exponent for eGFR as covariate for CL; ω_V1_, square root of inter-individual variance for V_1_; ω_V2_, square root of inter-individual variance for V_2_; ω_CL_, square root of inter-individual variance for CL; ω_Q_, square root of inter-individual variance for Q; σ, residual variability for power error; SE(%), percent standard error.

**Figure 3:**
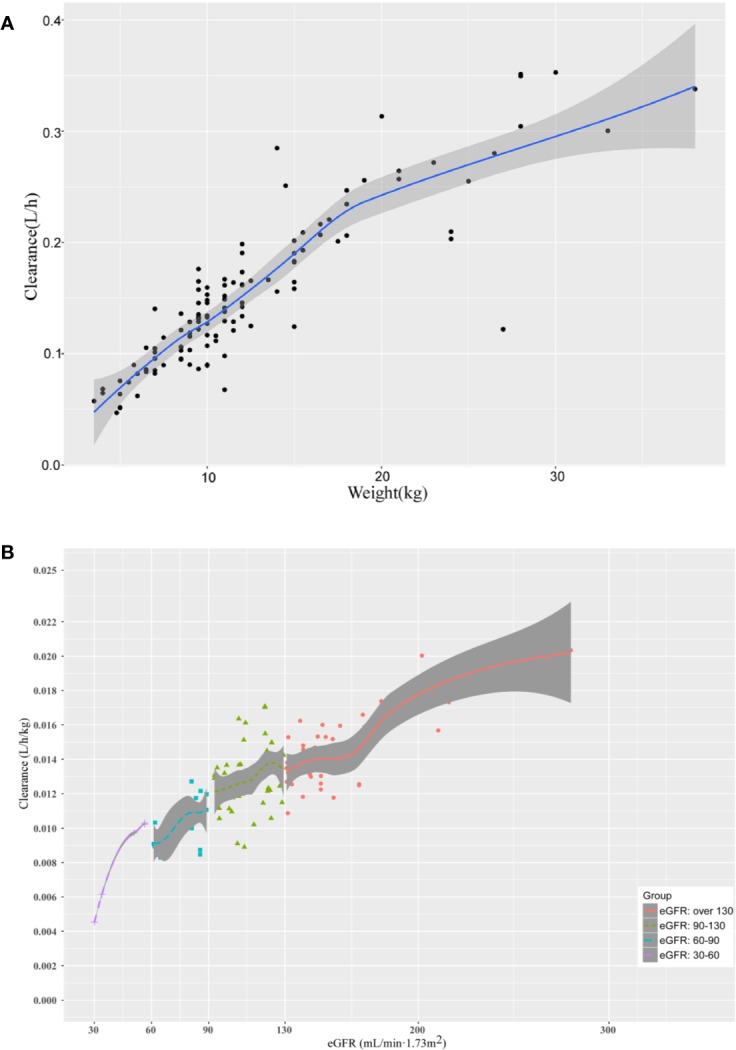
The relationship between **(A)** the CL of teicoplanin and WT; **(B)** the CL of teicoplanin and eGFR for children with augmented renal function, normal renal function, mild renal insufficiency and moderate renal insufficiency. The shaded areas indicate 95% CIs for the locally weighted scatterplot smoothing fit.

As shown in **Figure 3**, the relationship between defined covariates and CL was visualized by locally weighted scatterplot smoothing method. The results suggested that the CL of teicoplanin increased with WT and eGFR. In addition, eGFR of patients in the four different groups also positively correlated with the CL. As can be seen in **Table 5**, the results of variance analysis suggested that comparing to children with moderate or mild renal insufficiency, the weight-adjusted CLs of children with augmented and normal renal function were significantly higher (P < 0.001).

**Table 5:** Pharmacokinetic parameters of groups with various renal function status estimated with Bayesian method (n = 136, mean ± SD).

Group	N	V1 (L/kg)		V2 (L/kg)		CL (L/h/kg)		Q (L/h/kg)	
		Estimate	95%CI	Estimate	95%CI	Estimate	95%CI	Estimate	95%CI
eGFR ≥ 130	43	0.29 ± 0.51	0.13–0.45	1.36 ± 0.41	1.24−1.49	0.015 ± 0.002	0.014–0.015	0.016 ± 0.008	0.014−0.019
90 ≤ eGFR < 130	62	0.27 ± 0.24	0.21−0.33	1.79 ± 0.75	1.60−1.98	0.013 ± 0.002	0.013−0.014	0.023 ± 0.014	0.019−0.026
60 ≤ eGFR < 90	23	0.26 ± 0.16	0.19−0.33	1.71 ± 0.63	1.44−1.99	0.010 ± 0.001	0.010−0.011	0.022 ± 0.010	0.018−0.027
30 ≤ eGFR < 60	8	0.33 ± 0.32	0.06−0.60	2.28 ± 1.47	1.05−3.52	0.008 ± ± 0.003	0.006−0.010	0.027 ± 0.017	0.013−0.041
Total	136	0.28 ± 0.34	0.22−0.33	1.67 ± 0.74	1.55−1.80	0.013 ± 0.003	0.012−0.013	0.021 ± 0.012	0.019−0.023
P value		0.954		0.002		0.001		0.017	

eGFR is given in ml/min·1.73m^2^.

### Validation of Final Population Pharmacokinetics Model

**Figure 4:**
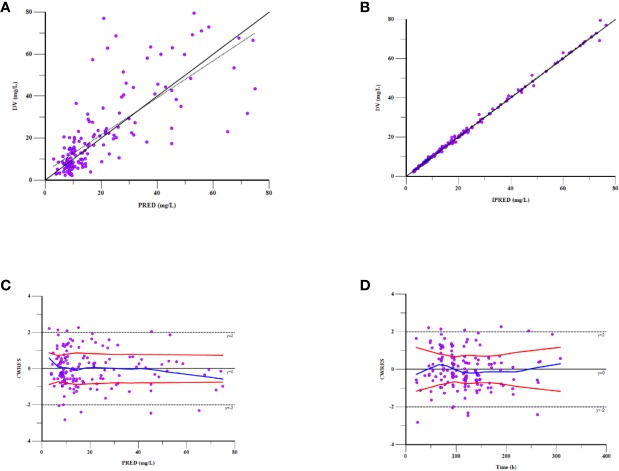
Goodness-of-fit plot for the final population pharmacokinetics model. Observations against **(A)** population predictions (PRED) and **(B)** individual predictions (IPRED); **(C)** CWRES against PRED; **(D)** CWRES against time after the last dose.

**Figure 5:**
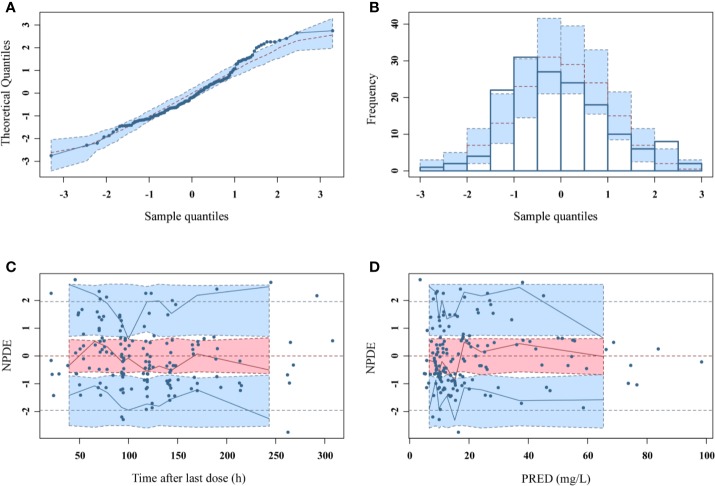
NPDEs of the final population pharmacokinetic model. **(A)** Quantile-quantile plot against the expected standard normal distribution; **(B)** Histogram of NPDE with the density of the standard normal distribution overlaid; **(C)** Scatterplot of NPDE against time; **(D)** Scatterplot of NPDE against PRED.

**Figure 6:**
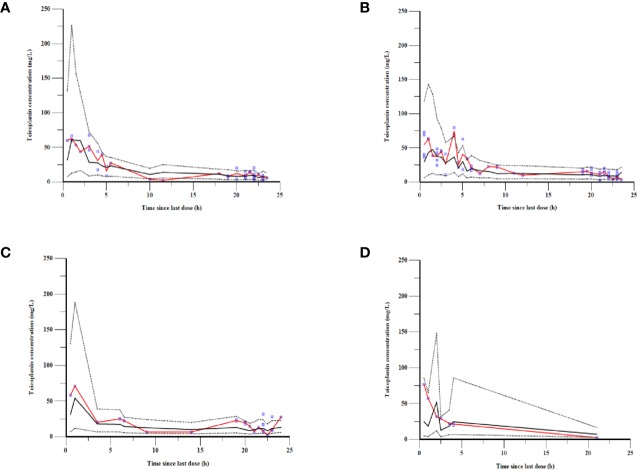
VPCs of the final model for children with **(A)** augmented renal function, **(B)** normal renal function, **(C)** mild renal insufficiency, and **(D)** moderate renal insufficiency. The blue points represent the observed value. The red lines are the median lines of observed concentrations. The dashed lines show the 2.5th and 97.5th percentiles and the solid line shows the 50^th^ percentile of the simulated data.

As can be seen in **Figure 4**, the goodness-of-fit plots showed that the observed plasma concentrations and the model predictions were closely agreement with each other, which suggested the predictive accuracy of the final model. Most of the concentration data were laid around 0 and within an SD of ±2 of the normalized units. As shown in **Table 4**, the parameter estimates of the population pharmacokinetics model distributed in the 95% CIs obtained from the nonparametric bootstrap procedure for 1,000 times. At the same time, the biases (< ± 10%) were acceptable between the parameter estimates and bootstrapped median parameter estimates, demonstrating the stability of the population pharmacokinetics model. As shown in **Figure 5**, the results of NPDE measured by t-test (P = 0.604), Shapiro Wilks test (P = 0.063), Fisher’s variance test (P = 0.083), and Global test (P = 0.190), suggested NPDE followed a normal distribution with variance homogeneity. **Figure 6** showed the VPCs of concentrations versus time in children with different renal functions. For both of the children with augmented, normal, and impaired renal functions, most simulations were laid within the 95% CI of prediction, which proved the predictive capability of the final model. In summary, the final population pharmacokinetics model presented good accuracy and stability and a predictive capability for individual and population pharmacokinetic parameters.

### Simulation and Dosing Regimen Optimization

**Figure 7:**
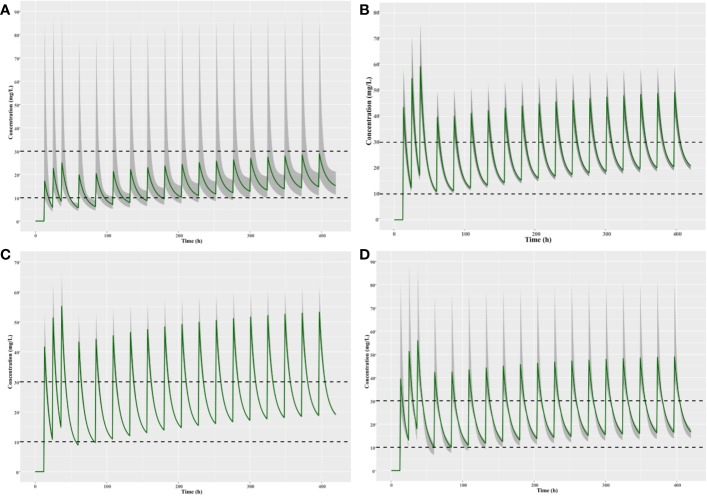
Simulation of different teicoplanin dosage regimens for children with **(A)** moderate renal insufficiency, **(B)** mild renal insufficiency, **(C)** normal renal function, and **(D)** augmented renal function. The shaded area represents the 95% CI of the final parameters.

The population pharmacokinetics parameters of patients with different renal functions were shown in **Table 5**. The results suggested that the CLs of teicoplanin in children with augmented and normal renal function were significantly higher than that of the children in the other two groups. As shown in **Figure 7**, optimal dosing regimens were simulated for children with different renal functions based on each pharmacokinetic parameter, aiming to achieve the target trough concentration of 10–30 mg/L. The results suggested that optimal dosing regimens for children with different renal functions were as follows: children with moderate renal insufficiency need three loading doses of 6 mg/kg q12h followed by a maintenance dose of 5 mg/kg qd; children with mild renal insufficiency require three loading doses of 12 mg/kg q12h followed by a maintenance dose of 8 mg/kg qd; children with augmented or normal renal function should be given three loading doses of 12 mg/kg q12h followed by a maintenance doses of 10 mg/kg qd.

## Discussion

Teicoplanin is a glycopeptide antibiotic for the treatment of patients infected by Gram-positive bacteria. Due to its wide use in children recently and the significant differences in renal function between pediatric patients, pharmacokinetic variability is significant. In addition, teicoplanin has a long elimination half-life, so it needs long time to achieve the steady-state concentration. Therefore, the loading doses are required to quickly reach a steady-state serum concentration.

However, researches on pharmacokinetic study and dosage regimen of teicoplanin in Chinese children are limited. The purpose of our study was to establish the population pharmacokinetics model of teicoplanin in Chinese children with different renal functions, which was employed to define teicoplanin pharmacokinetic parameters and quantify the influence of clinical and demographic factors on teicoplanin PK characteristic. The results of the population pharmacokinetics studies suggested that a two compartment model with first order elimination was the best fit, accompanying with both weight and eGFR being significant covariates. In our work, the mean CL of teicoplanin was 0.013 L/h/kg in Chinese children aged 0–10 years. Wei Zhao et al. reported that the CL of teicoplanin was 0.015 L/h/kg in French children with malignant haematological disease aged 0.5–16.9 years, weighting 7.7 to 90.6 kg (Zhao et al., 2015). In addition, Martin et al. also reported a mean CL of 0.019 L/h/kg in children aged 2–11 years with average weight of 21.18 kg (Ramos-Martín et al., 2014).

Both weight and eGFR explained significant portions of the variance of CL or V_1_ after covariate screening procedure and were included into the final population pharmacokinetics model. During the covariate screening process, we found that the body weight impact on V_1_ presented as natural log transformed WT could reduce the OFV to a greater extent. As can be seen in **Figure 3**, the CL increased with eGFR after accounting for the variances of WT by employing the estimated exponent allometric relationship. The results of our work suggested that the CL of teicoplanin was positively correlated with eGFR, which was consistent with previous studies (Yamada et al., 2012; Zhao et al., 2015). Ramos-Martín et al. reported that the weight of children affected CL *via* linear and allometric scaling terms (Ramos-Martín et al., 2014). However, they did not investigate the relationship between eGFR and teicoplanin CL. In 2017, Ramos-Martín et al. again investigated the relationship between the teicoplanin CL and weight, age, and eGFR in neonates and children. They found that the relationship between teicoplanin CL and weight was apparent and there was an exponential relationship between eGFR and CL in children over 3 months old, which is in consistent with our study. At the same time, they also found that teicoplanin CL was significant related to the postnatal age and serum creatinine concentration in infants younger than 3 months (Ramos-Martín et al., 2017). Due to the limited cases of children less than 3 months in our study, we did not find such a correlation.

Teicoplanin had a time-dependent killing pattern, so the value of AUC_24_/MIC would be better to predict its antimicrobial capacity (Ogawa et al., 2013). However, the target value of AUC_24_/MIC for teicoplanin did not have been extensively studied. Clinical efficacy also could be assessed by the teicoplanin trough concentration (Harding and Sorgel, 2000; Kobayashi et al., 2016), and the trough concentration of 10–30 mg/L had a guarantee for most infections as previously reported. In order to reach the trough level, children with different renal functions required different loading and maintenance doses because of their different teicoplanin CL. An elevated dose for 0–10 years old children with augmented and normal renal function should be increased as the loading dose of 12 mg/kg q12h followed by a maintenance dose of 10 mg/kg qd. Ogawa et al. also suggested that it is beneficial for the clinical outcomes by increasing loading doses (Ogawa et al., 2013). As Sato et al. reported, lacking of loading dose may cause a significant teicoplanin underexposure in the early therapy period (Sato et al., 2006), which were consistent with our study. The previous reports suggested that elevated serum creatinine level would significantly increase when the trough concentration of teicoplanin >60 mg/L (Tobin et al., 2010; Lemaire et al., 2011; Strenger et al., 2013). According to the previous studies, when the trough concentration of the teicoplanin was over 30 mg/L, the development of hepatic function disorders would be more frequent (Nakamura et al., 2015). Wilson suggested that an increased risk of organ toxicity would occur if teicoplanin trough concentration was greater than 40 mg/L (Wilson, 1998). Teicoplanin trough concentration less than 30 mg/L is suggested to be safe for patients infected with Gram-positive bacteria (Zhou et al., 2018). We did not find researches reporting the relationship between teicoplanin peak concentration and clinical safety. According to our clinical observations, no significant adverse reactions were found in patients with peak concentrations of 70–80 mg/L. Based on this observation, the simulated optimal dose regimens aimed to restrict Cmax to be less than or not too much over 80 mg/L while the steady-state Ctrough to be within the range of 10–30 mg/L for all patients with different renal functions, taking into account the clinical efficacy and safety.

The limitations of our work are as follows: (i) lack of clinical data to define the therapeutic effect; (ii) in view of the small sample size of the moderate renal insufficiency group and the large variability of pharmacokinetic parameters, it is recommended that the blood drug concentration should be closely monitored for these patients; (iii) the safety and efficacy of the optimized teicoplanin dosing regimens was just simulated based on safety and efficacy from previous studies but has not been actually verified.

## Conclusion

In our study, the population pharmacokinetics of teicoplanin in children of age 0–10 years with different renal functions was investigated. Children with different renal functions demonstrated different teicoplanin CLs. Therefore, different dosing regimens were required for them for optimized clinical outcomes. Finally, we suggested the optimal dosing regimens of teicoplanin for children with different renal functions. The final population pharmacokinetics model provides a useful tool for teicoplanin dose individualization as it estimated individual pharmacokinetic parameters of children with different renal functions. However, further studies are necessary to evaluate the safety and therapeutic effects of the optimized dosage regimen proposed by this study.

## Data Availability Statement

The raw data supporting the conclusions of this article will be made available by the authors, without undue reservation, to any qualified researcher.

## Author Contributions

LG performed the major research. The manuscript was written mainly with the efforts of LG and YW. The clinical materials were collected by TK. CC and YW provided the statistical analysis. JW, QY, SL, YM, and CN have given approval to the final version of the manuscript. HX contributed as consultant.

## Conflict of Interest

The authors declare that the research was conducted in the absence of any commercial or financial relationships that could be construed as a potential conflict of interest.

## Supplementary Material

Supplementary Figure 1 Correlation analysis on all of the covariates.
