# Supplementary figures and images for: Population Pharmacokinetics and Dosage Optimization of Teicoplanin in Children With Different Renal Functions
Source: Front Pharmacol. 2020 May 5;11:552. doi: 10.3389/fphar.2020.00552 (PMC7214819; doi:10.3389/fphar.2020.00552)

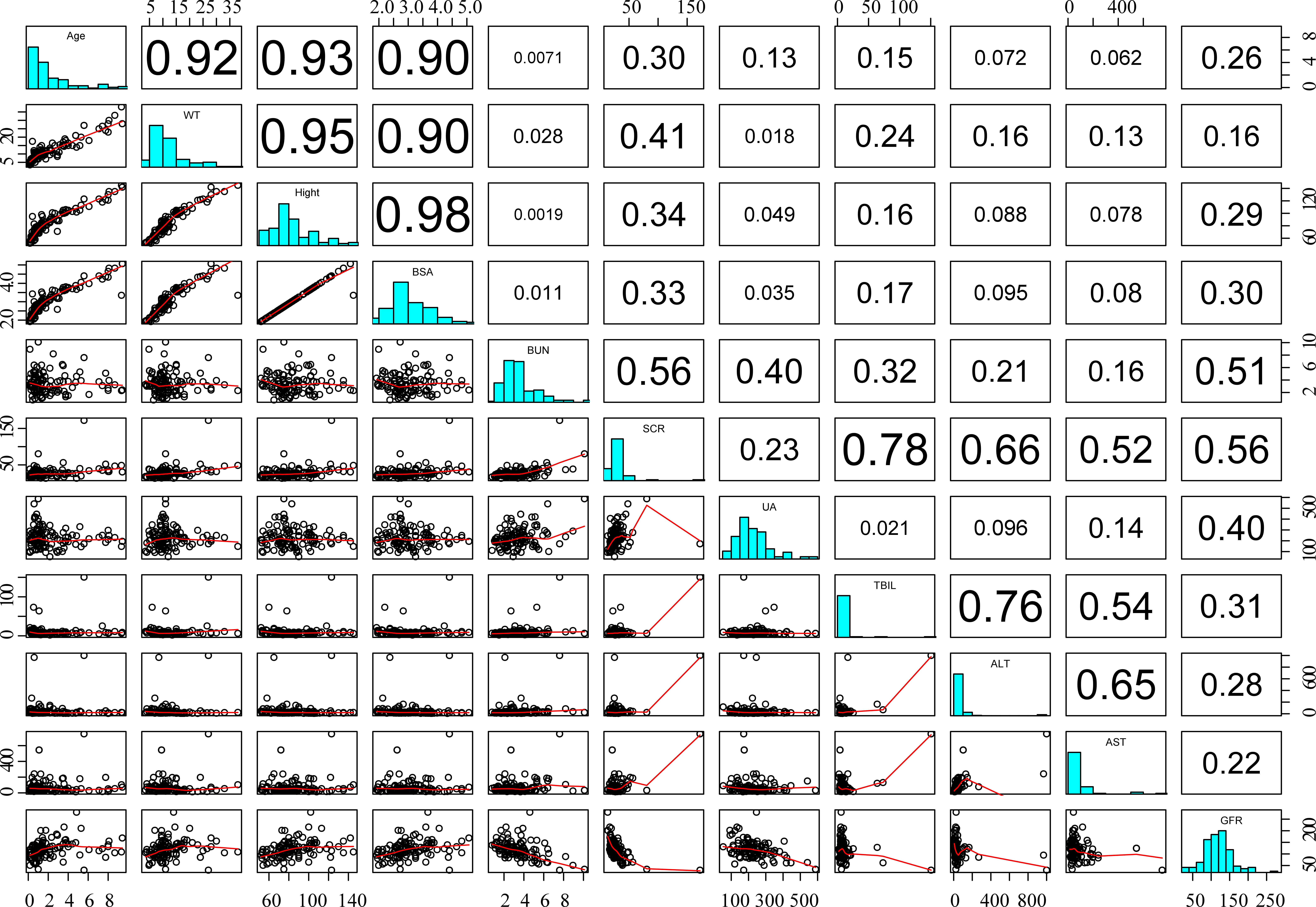

Supplement: Supplementary Figure 1 — Correlation analysis on all of the covariates. [file Image_1.tif]
